# Identifying the Branch of Kiwifruit Based on Unmanned Aerial Vehicle (UAV) Images Using Deep Learning Method

**DOI:** 10.3390/s21134442

**Published:** 2021-06-29

**Authors:** Zijie Niu, Juntao Deng, Xu Zhang, Jun Zhang, Shijia Pan, Haotian Mu

**Affiliations:** College of Mechanical and Electronic Engineering, Northwest Agriculture & Forestry University, Xi’an 712100, China; dengjuntao@nwafu.edu.cn (J.D.); zx0825@nwafu.edu.cn (X.Z.); junzhang@nwafu.edu.cn (J.Z.); pan21@nwafu.edu.cn (S.P.); 635406182@nwafu.edu.cn (H.M.)

**Keywords:** deep learning, unmanned aerial vehicle, kiwifruit, image segmentation

## Abstract

It is important to obtain accurate information about kiwifruit vines to monitoring their physiological states and undertake precise orchard operations. However, because vines are small and cling to trellises, and have branches laying on the ground, numerous challenges exist in the acquisition of accurate data for kiwifruit vines. In this paper, a kiwifruit canopy distribution prediction model is proposed on the basis of low-altitude unmanned aerial vehicle (UAV) images and deep learning techniques. First, the location of the kiwifruit plants and vine distribution are extracted from high-precision images collected by UAV. The canopy gradient distribution maps with different noise reduction and distribution effects are generated by modifying the threshold and sampling size using the resampling normalization method. The results showed that the accuracies of the vine segmentation using PSPnet, support vector machine, and random forest classification were 71.2%, 85.8%, and 75.26%, respectively. However, the segmentation image obtained using depth semantic segmentation had a higher signal-to-noise ratio and was closer to the real situation. The average intersection over union of the deep semantic segmentation was more than or equal to 80% in distribution maps, whereas, in traditional machine learning, the average intersection was between 20% and 60%. This indicates the proposed model can quickly extract the vine distribution and plant position, and is thus able to perform dynamic monitoring of orchards to provide real-time operation guidance.

## 1. Introduction

In kiwifruit orchards, the acquisition of the plant growth status is particularly important for managers, and can help reduce the cost of orchard management and improve the efficiency of resource utilization. With the support of 3S (RS, GPS, GIS) technology, accurate remote sensing information of the orchard vegetation can be obtained, such as plant location and coverage [1]. Diversified information obtained via online methods enables managers to more accurately understand the growth of plants and control the spraying of pesticides [2], and make correct judgments regarding fertilization, pruning, and harvesting. Therefore, there is an urgent need to develop a fast, nondestructive, and stable technology to obtain plant information in kiwifruit orchards.

The unmanned aerial vehicle (UAV) is a new low-altitude remote sensing platform that can provide ultra-high-resolution images, flexibility for planning according to the weather and other factors, and dynamic remote sensing information [3]. Due to their high resolution and real-time characteristics, UAVs are a popular technology for obtaining large-scale plant information from UAV images. Different sensors that are carried by UAVs have been used to identify and count trees [4], and to determine the tree height and crown size [5,6]. This form of information collection, which is characterized by low data accuracy, is often used in the field of forestry management, which does not require accurate management of each tree. However, for the fine management of orchards, knowledge regarding the distribution of the plants is required, and the position and growth of each plant must be monitored, Thus, it is necessary to use more accurate sensors and more accurate plant phenotype processing algorithms. In recent years, the combination of low-altitude UAV remote sensing data, and deep learning data collection and processing, has significantly promoted the detailed management of orchards. For example, to count the number of citrus trees in an orchard [7], a simple CNN was designed and implemented. Based on the high-resolution images collected by the UAV, the CNN, which was a target recognition method, was able to separate citrus trees from other tree species. In orchard remote sensing, only simple parameters of trees, such as the crown size, can be obtained. To obtain more abundant information about the fruit trees from remote sensing images, Ampatzidis et al. extracted the number, crown size, and health index of fruit trees [8]. At present, the plant growth status obtained by UAV is often limited to the flourishing period of the plant. Thus, a valuable research area is the identification of changes in plants’ annual growth using UAV remote sensing, and the linking of these changes. In the plant growth model established in this study, UAV real-time images were employed to judge the growth status of plants at any time of the year. Subsequently, the labor intensity was significantly reduced, and errors arising from manual judgment could be avoided.

To date, few reports have been published regarding the extraction of branches. Wu et al. [9] proposed an extraction method for the crowns of bare trees. The method used the target recognition and segmentation of the CNN to realize the extraction and calculation size of a bare apple tree crown. Although this method was mainly focused on trees, the branches of apple trees are shorter and stronger, and artificial trellises are not used to fix them. Actinidia is a kind of liana that has a slender stem and needs to grow on scaffolds. Trellis planting is a common planting method. It is difficult to determine the plant position and canopy distribution in closed orchards when the fruit are maturing. Obtaining the distribution of the vines in advance is helpful for predicting the growth and guidance of the pruning. However, analyzing the vines of lianas is usually more challenging than analyzing the branches of trees. This is because: (1) the stems of the lianas are slender and irregular; (2) steel wires and slender stems are mixed among the scaffolds, which makes it difficult for human experts to accurately distinguish them; (3) and the ground is covered by grass, the background is not clear, and the texture is complex. The above reasons result in a diverse and misleading pattern for kiwifruit vine segmentation.

This study proposes an effective plant location distribution model (PLD-M) to interpret kiwifruit remote sensing images obtained using a low-altitude UAV. The three algorithms constitute the basis of the kiwifruit vine data acquisition and analysis model. First, according to the YOLOv3 deep learning target recognition network [10,11], the kiwifruit trunk in the UAV image is recognized and located. The improved version of this algorithm is also often used in the recognition of small targets in the agricultural field, such as pinecones [12], cherries [13], and apples [14]. The second key method, PSPnet [15], is a residual CNN used to extract the kiwifruit vines from the background using semantic segmentation. The third key approach, used to resample the region with vine pixels from the semantic segmentation results, provides the possible coverage and coverage gradient of the kiwifruit canopy. Next, the distribution of the kiwifruit vines and plants in the field is obtained by combining the location and gradient information. The specific objectives of this study include the following: (1) Compare and explore the advantages and potential of the kiwifruit field location and distribution models that are based on the low-altitude remote sensing images and deep learning modeling, in comparison to the support vector machine (SVM) and random forest (RF) classifiers. (2) To compare the effects of the different thresholds and sampling sizes on the PLD-M intersection over union (IoU). (3) To provide a fast and accurate method to obtain kiwifruit plant information in orchards. To verify the effectiveness of the PLD-M, a training set and a plant test set with 600 calibration images and 40 high-definition images, respectively, were constructed. We have published the dataset on GitHub: https://github.com/eletricsheep/PLD-M/tree/main. Last accessed at 23 June 2021.

To establish PLD-M model, the collected data were processed as follows:

(1) The images were spliced by Pix4D to generate an orthophoto;
(2) The orthophoto image was clipped to the size suitable for network training, and 300 images were selected to make the data set;
(3) Three kinds of classifiers were trained by data set, and the trained classifiers were used to process the image to obtain the segmented image;
(4) Different sampling size *S* and threshold level T were set to process the segmented image and count the IoU;
(5) According to the change of IoU, the best parameters of *S* and T were selected to test 40 test images to verify the performance of the model;
(6) Finally, the PLD-M model was formed.

## 2. Materials and Methods

### 2.1. Experimental Sites

**Figure 1:**
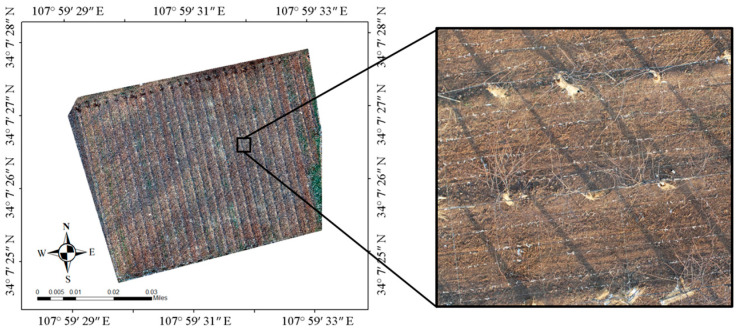
Remote sensing image of the experimental land and field enlarged picture.

The experimental site is located at the kiwifruit experimental station of Northwest Agricultural and Forestry University, Meixian County, Baoji City, Shaanxi Province, China (107°59′31.4443″ N, 34°7′27.8819″ E, elevation 643.22 m). The plant varieties in this experiment site were Heywood, with tree ages of 5–6 y, a plant spacing of 3 m, and a row spacing of 4 m. The area of the collection experiment site was 8814 m^2^ (Figure 1). To obtain a clear image of bare vines, the UAV images were collected at 14:32 on 14 January 2021, local time; the leaves of the plant had shed completely, and residual branches were left around the plant.

In this study, four rotor UAV (DJI phantom 4pro V2.0) was employed to aerial photography. Due to its short endurance and instability [16], both the course and side overlap were set at 80%. Table 1 shows the aerial photography and image parameters.

The UAV took 434 remote sensing images in total. The original orthophoto images were obtained by Pix4dmapper and mosaic with the CS_WGS_1984 coordinate system.

### 2.2. Brief Introduction of the PLD-M

**Figure 2:**
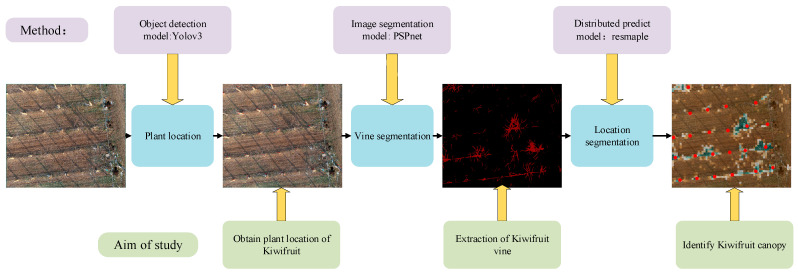
Flowchart of the kiwifruit location and distribution prediction. The orchard image is used to recognize a single tree. The vine is separated from the background through image segmentation to prepare for the next step of predicting the distribution. A two-dimensional map with plant location and canopy distribution information is formed.

The PLD-M can be divided into three phases, as shown in Figure 2. In the first stage, the position of the kiwifruit in the orchard was determined. During this stage, the most important task was to identify and calibrate the main position of the kiwifruit plants using target recognition. Therefore, we constructed a kiwifruit plant target detector using YOLOv3 (the network has an excellent small-scale target recognition performance). Then, we obtained the number of kiwifruit plants, the location of each tree, and the spatial distribution of each tree in the UAV image. The range of the vine pixels in the image was extracted. This study compared the performance of PSPnet, SVM, and RF to obtain the optimal algorithm as the segmentation model of the vines, and separate the vines from the background. Finally, the entire image was resampled and normalized based on the vine pixel data. The ratio of the vine pixels was returned to each sampling size, and spliced into the distribution map with gradient information and the location image that was obtained in the first stage. Finally, the location distribution of the output plants was determined. The PLD-M integrates the detection, segmentation, and distribution range prediction of the plants. Therefore, it is possible to extract more information from the UAV images, such as the number, location, and position of vines, and the possible canopy distribution range. The PLD-M is relatively independent in each stage, but each stage is necessary to complete the canopy prediction. Details of the three stages are given in Section 2.3 and Section 2.4.

### 2.3. Extraction of the Kiwifruit Vine

#### 2.3.1. Random Forest Image Segmentation

The RF classifier, an ensemble-learning algorithm, was first proposed by Leo Breiman and Adele Cutler in 1995. The RF classifier contains multiple decision trees, and its output category is determined by the mode of the output category of the individual trees. As a result, it can generate a classifier with high accuracy for many kinds of input data. Because there is no complex parameter adjustment, it is widely used in remote sensing image classification, such as crop nitrogen content estimation [17] and land use classification [18].

#### 2.3.2. Support Vector Machine Image Segmentation

SVM [19], a class of generalized linear classifiers, is a supervised learning method for the binary classification of data memory. The related theories and problems of SVM were first proposed in 1964, and a series of improved algorithms were rapidly developed in the 1990s. SVM is based on the principle of structural risk minimization, which has many advantages: it can avoid overlearning problems, has excellent generalization ability, is superior to other algorithms in terms of small samples and unbalanced data sets results [20], has a fast classification speed, and the results are easy to explain. This algorithm is also often used in the detection of agricultural diseases [21], pests [22], and other fields [23].

#### 2.3.3. Deep Semantic Segmentation

**Figure 3:**
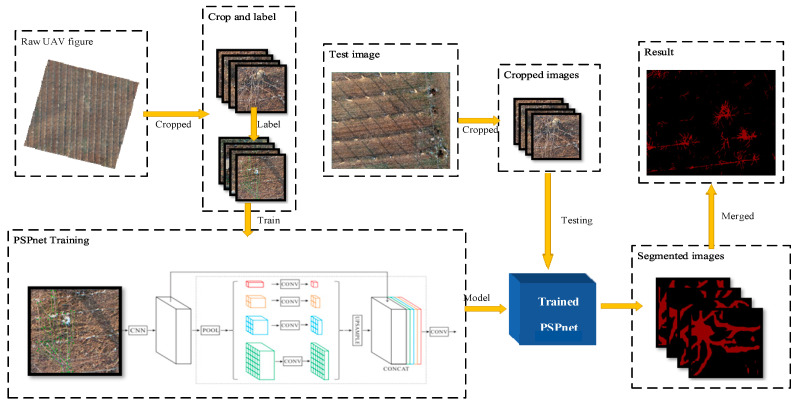
The original UAV image is clipped and labeled, and then input into the PSPnet network for training, and the training model is obtained. The image of the test set is clipped and input into the model to obtain the segmented image. Finally, the segmented image is stitched to obtain the segmented image of the remote sensing image.

The pyramid scene parching network (PSPnet) is an effective optimization strategy for deep Resnet [24] development, which monitors the change in the loss value. Because of the low position information of the high-level features in a deep network, it improves the content of the high-level features using a special fusion of the multi-scale features, and then uses a conditional random field (CRF) to process the segmentation results. PSPnet divides the full revolutionary network (FCN) images into four scales (1 × 1, 2 × 2, 3 × 3, 6 × 6) and then adds them to the input image to improve the prediction accuracy. The training and testing processes of the model are illustrated in Figure 3.

The calibration image of the dataset was labeled with the image after cutting 417 × 417 pixels. The spatial resolution of the image was 0.02 m, and the label file was made with Labelme. A total of 300 calibration images were amplified and input into the network for training. The experiment was performed on a Windows 10 professional operating system (Microsoft). This was achieved by using the pytorch1.7.1 framework that was built by Anaconda 3.0, and we built the network under Python 3.6.10 and Cuda11.1 to accelerate training. The details of the equipment are listed in Table 2.

To optimize and train the PSPnet deep semantic segmentation network, a training cycle of 100 generations was established and split into two stages, with a different Batch_size and Learning_rate. The specific training parameters are presented in Table 3.

We established two stages for the training of the model. The first stage aimed to improve the training speed, accelerate the convergence of the model; the learning rate was set as $10^{-4}$ and batch_size was eight. In the second stage, to identify the optimal solution, the learning rate was set to $10^{-5}$ and batch_size was four. For this study, training was conducted on VOC2007, which has two categories: background and kiwifruit vine. To reduce the gap between the predicted pixel class and the expected pixel class, we used the cross-entropy Function (1) to train the model:

(1) $$L(p,q)=-\sum_{i}\sum_{n}p_{in}\log q_{in}$$

where *p* and *q* represent the expected pixel category and the predicted pixel category, respectively; *i* is the pixel; *n* is the category; $p_{in}$ and $q_{in}$ represent whether pixel *i* is classified in *n* (1 if classified into *n*, 0 if not). The classification accuracy index of the image was measured by the mean average precision (mAP). This represents the average value of all of the categories, as a percentage, i.e., the ratio of the number of correctly classified pixels of each type to all of the categories, as in Equation (2):

(2) $$mAP=\frac{1}{n_{c}}\sum_{i}\frac{p_{i}}{\sum_{j}p_{j}}$$

where $n_{c}$ represents the number of all the categories and $p_{j}$ represents the number of class *i* pixels that are predicted as class *j*.

### 2.4. Plant Location and Distribution

#### 2.4.1. Resampling Processing

After the segmentation algorithm, the pixels containing the vines in images were demarcated; however, because the distribution of the vines is divergent from the center to the edge, only the discrete pixels of the vines can be obtained from the images. This results in a large amount of “pepper and salt noise” after classification.

**Figure 4:**
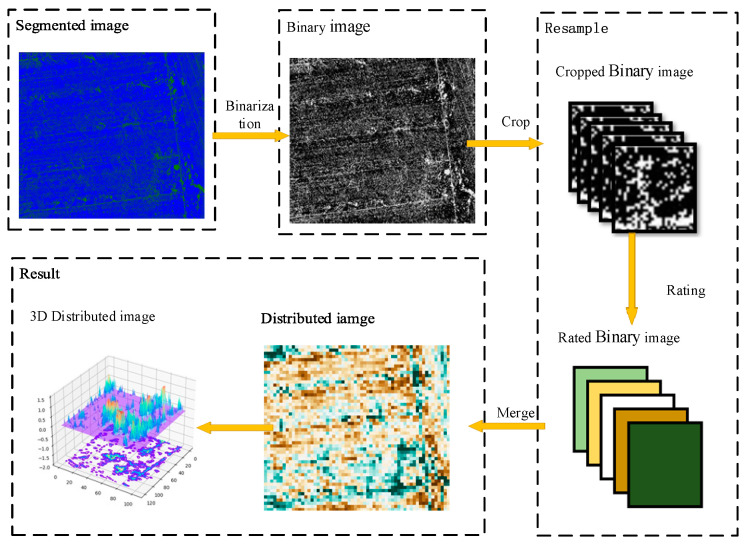
Flowchart of resampling the normalization algorithm shows that the green and blue pixels in the segmented image are the kiwifruit vine and ground, respectively. After binarization, the image is clipped with multiple sampling sizes, and the proportion of the kiwifruit pixels in each image is calculated. Then, the color is filled according to the gradient order. Finally, the vine distribution map is generated.

To address the problems discussed above, firstly, the remote sensing image was binarized using three classification methods. Secondly, the binary clipping image was clipped according to the sampling size; the sampling size was represented by the letter *S*, and the proportion of vine pixels was counted. Finally, the resampled image was reconstructed by gradient filling. In this method, the interference pixels with large sparse distribution were screened out, and the influence on the distribution prediction was reduced. The resampling normalization process is shown in Figure 4.

The sampling size *S* is shown as:

(3) S=(35,40,45,50,55,60,65,70,75,80,85,90)

During resampling normalization, we can take the number of pixels in a single channel image as the denominator of the normalization, and the ratio of the normalized pixels is expressed by the letter *R*, as shown in Equation (4):

(4) $$R=\frac{p_{i}}{p_{i}+p_{j}}$$

where $p_{i}\;\mathrm{and}\;p_{j}$ represent the ratio of pixels that are divided into kiwifruit vines and the ground class, respectively, which reflects the proportion of the vine to the cut image.

#### 2.4.2. Coordinate and Resampling

Using the trained YOLOv3 network, the main stem of the kiwifruit was identified and selected from the image. YOLOv3 is a target recognition network based on Darknet_53; it is a full convolutional network (FCN) with 53 convolution layers. Compared with other target recognition networks, the YOLOv3 network has three scale outputs (13 × 13, 26 × 26, 52 × 52) in the down-sampling process, which makes it more effective for different sizes of targets, and particularly small targets. To distinguish the bare land area and the vine area more clearly, the gradient map palette uses the Seaborn Python data visualization library. After overlapping and comparing the images, the vine density was divided into four levels, as shown in Table 4.

**Figure 5:**
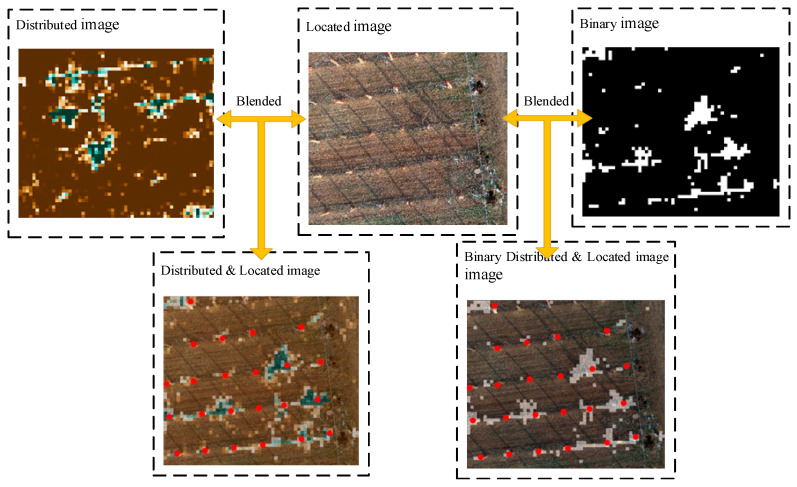
By fusing the resampling distribution image, the binarization distribution image, and the target recognition location image, the binarization location distribution image and the location distribution image are obtained.

The YOLOv3 network outputs the image with the kiwifruit plant position (the plant position is the red box calibration; to enhance the display, this study conducted the secondary annotation through the red dot), and then fuses the image after semantic segmentation and resampling. The fusion process for the location and distribution images is shown in Figure 5.

## 3. Results

### 3.1. Analysis of the Image Processing Results

#### 3.1.1. Accuracy Evaluation of the RF, SVM, and PSPnet

After image segmentation, we obtained the segmentation images of the RF, SVM, and deep semantic segmentation. According to the calculation, we obtained the mAP and signal-to-noise ratio (SNR) of each classification method, as shown in Table 5.

**Figure 6:**
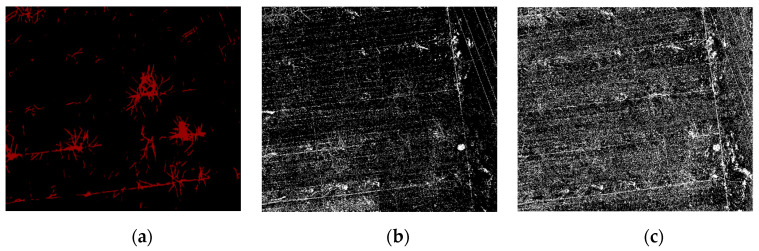
Classification effect picture under the different classification methods. As demonstrated, (**a**) is the result of the PSPnet classification, (**b**) is the result of the SVM classification, and (**c**) is the result of the RF classification.

By comparing the mAP of the three segmentation methods, it can be observed that the pixel accuracy of SVM was approximately 85%. The accuracy of PSPnet was 71%, i.e., 14 percentage points lower than that of SVM. From the perspective of this data, the mAP of the two traditional machine learning segmentation methods is higher than that of deep semantic segmentation. An image of the classification result is shown in Figure 6.

**Figure 7:**
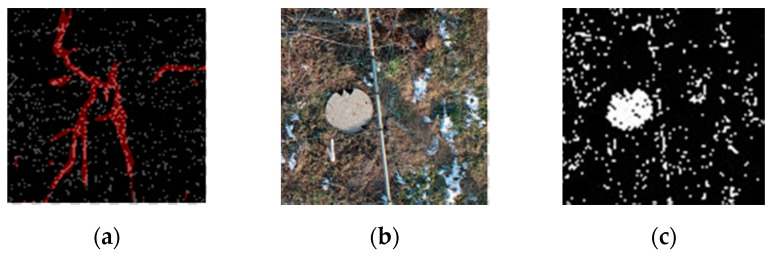
Comparison of the deep semantic segmentation and traditional machine learning segmentation. (**a**) The red part is the classification result of PSPnet, and the white pixels represent the classification effect of the SVM. It can be observed that the red pixels are more continuous and concentrated, and there are many surrounding white pixels. (**b**) The well covers and scaffolds in the UAV image. (**c**) The SVM mistakenly identifies irrelevant objects.

The comparison of the segmentation results in Figure 6 shows that the noise for traditional machine learning is greater. The RF and SVM are more sensitive to the fine features than the depth semantic segmentation, and the distribution range of the vine pixels in the correct region is more accurate. However, in general, the quality of the segmentation image in traditional machine learning is not as high as that obtained by deep semantic segmentation. There are three reasons for this finding. First, the pixels obtained from machine learning are separated from each other, which results in a mixture of pixels in the background, the object, and a rough outline of the object edge. In addition, there is more noise at the edges. Second, because of the pruning that takes place in winter, there are more residual branches on the ground and more complex ground objects. The texture of the UAV image obtained in practice is more complex. The RF and SVM incorporate pixels that are similar to the label into the target object, which further increases the noise of the segmentation results. Finally, various objects (e.g., well covers, wires, and scaffolding wires) and the objects on the edge of the orchard are similar to the target objects. As demonstrated in Figure 7a, in comparison to machine learning, due to the integrity of the dataset annotation, deep semantic segmentation does not segment the ground stumps as objects. Furthermore, the segmented objects have a continuous pixel distribution, smooth contour, and no noise. They also do not include the objects at the edge of the orchard, such as well covers and scaffolding, in the objects, as shown in Figure 7c.

#### 3.1.2. Advantages of Deep Semantic Segmentation

The average pixel accuracy of PSPnet is lower than that of the former two traditional machine learning segmentation methods. However, in the actual classification results, the results that are obtained by deep semantic segmentation have a better continuity than in traditional machine learning, and the recognition of objects is also better than that of traditional machine learning. In contrast to previous methods of remote sensing deep learning (such as field [25], citrus tree [26,27], and orchard remote sensing), kiwifruit vine recognition presents unique characteristics. First, because of the high density and uniform crops that are planted in the field, the target objects need to be recognized in the field crop images, which generally have continuous and uniform features and an obvious texture. In the process of kiwifruit vine recognition, the background pixels generally have an obvious texture, but the target objects are extremely small and difficult to distinguish. Second, compared with the larger and obvious branches of large fruit trees, kiwifruit trees are smaller and irregular.

The training of deep learning is robust. Due to the particularity of the features of kiwifruit orchards, PSPnet mainly avoids the misclassification of pixels. In the process of compiling the dataset, the range of vine pixels is relatively small, which makes it difficult to label, resulting in different error pixels in the labeled image. This small number of error pixels results in significant changes in the segmentation accuracy of traditional machine learning. However, when training in the PSPnet network, although it is difficult to label the vine pixels, these wrong pixels do not significantly affect the segmentation accuracy. The training time of the deep semantic segmentation network continues to increase with the increase in the sample size, and is relatively stable.

### 3.2. Influence of Threshold Parameters on the Accuracy of the Distributed Images

To evaluate the image distribution accuracy, due to the existence of the resampling size we can use the Otsu iterative thresholding method and other methods to select the segmentation threshold for a single time. In this study, the binary segmentation of each sample size *S* was performed for eight scales. A total of 288 binary images were obtained. The threshold value ranged from 0 to 1, and the eight gradients were equally divided, as indicated by the letter T. The corresponding relationship between the threshold and threshold levels is shown in Table 6.

**Figure 8:**
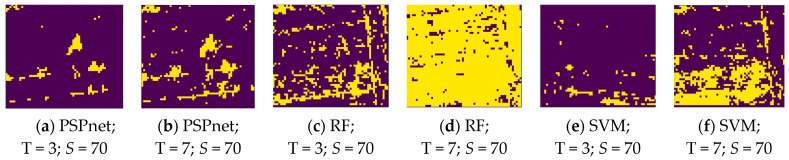
Binary images obtained by the different thresholds under three classification methods.

The distribution range of the real vines was compared to the continuous vines. The actual distribution range of the vines and the distribution range of the vines that were obtained after resampling were combined to calculate the IoU. The formula for the IoU is as follows:

(5) $$IOU=\frac{P\cap Q}{P\cup Q}$$

where *P* is the pixel range of the true vine distribution, and *Q* is the pixel range of the true vine distribution. The distribution map of the vines was binarized in the process of the IoU calculation, as shown in Figure 8. This is the binary image of the various classification methods under the *S* = 70 sampling size, and the T = 3 and T = 7 threshold levels.

Figure 8 shows the binary images of the three segmentation methods under the threshold levels of *S* = 70, and T = 3 and 7. The changes caused by the three different classification methods under the same parameter show that the pixel range of PSPnet segmentation is more concentrated, followed by that of SVM segmentation. As shown in Figure 8a,b,e, the pixels obtained by these two methods are more concentrated and show an island shape; the worst effect is that of RF segmentation. At a lower threshold level, the pixels are more discrete, such as in Figure 8c, but with the increase in threshold level, the useful information has been obscured by noise; Figure 8d. At the threshold level of T = 7, PSPnet can still maintain a relatively independent island distribution, whereas the SVM segmentation method results in increased noise. The reason for the above results is that, with the increase in the threshold level (that is, with the decrease in the threshold value), more pixels are allowed to be collected, which not only increases the number of useful pixels, but also increases the noise. Because the image SNR produced by the PSPnet segmentation method is better than that of the other two machine learning methods, due to the improvement in the threshold level, RF and SVM add a large amount of noise, whereas the pixel change of PSPnet is relatively stable.

**Figure 9:**
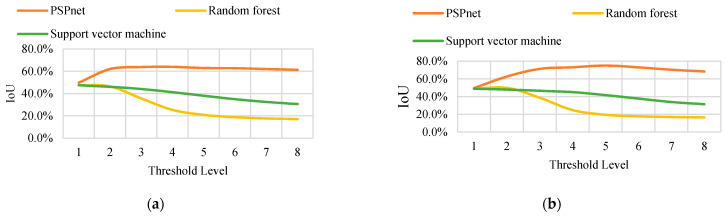
(**a**) The IOU curve of *S* = 35 obtained by the three classification methods changes with the threshold. (**b**) The IOU curve of *S* = 70 obtained by the three classification methods changes with the threshold.

In conclusion, the distribution of the pixels in the binary image shows that, with an increase in the threshold level (and thus a decrease in the threshold value), the binary image contains more information. The area of the yellow region (vine distribution region and noise region) changes with the threshold value. A higher threshold value can obtain continuous and low-noise binary segmentation results, but may also cause the original correctly predicted segmentation. From this, the layout area is removed, and the IoU of each binary image is calculated under eight threshold levels. The trend of the IoU variation with the accuracy is the same for the different sizes. Only the variation curves of the IoU with the threshold level T under the sampling sizes *S* = 35 and *S* = 70 are listed in the text, as shown in Figure 9. (Detailed data are shown in Table A1, Table A2, Table A3, Table A4, Table A5, Table A6, Table A7, Table A8, Table A9, Table A10, Table A11 and Table A12. Only the data in A1 and A9 are shown here).

The experimental data show that regardless of the resampling parameters, the order of the IoU that is obtained by the three classification methods from large to small is PSPnet, SVM, and RF classification. When the threshold level T = 5, the maximum IoU is obtained using PSPnet. At a small threshold level, the difference in the IoU that is obtained by the three segmentation methods is not obvious. However, with an increase in the threshold level, the IoU curves that are obtained by the three segmentation methods exhibit two trends. The IoU curve of the deep semantic segmentation shows a growing trend and remains stable after reaching the maximum IoU, and changes little with the threshold. The change curves of the two traditional machine learning methods show a monotonic decreasing trend, and the accuracy of the RF classification declines more rapidly.

There are two reasons for the above results. First, because the result of the depth network segmentation has continuous pixels and less noise distribution, the gradient of the pixels in the different regions of the resampled image is different. Even at a higher threshold level, it is still easy to extract different pixels in the resampled image. Second, traditional machine learning has some disadvantages in distinguishing complex textures and similar objects. Even through median filtering, it still cannot obtain good results (vine pixels and noise are similar in size, smaller filter cores cannot remove obvious noise, and larger filter cores will lead to the loss of vine pixel information). Therefore, when the threshold level increases, the mixed background noise is also included, which leads to an enlargement of the union area and a decrease in the IoU.

### 3.3. Model Evaluation

**Figure 10:**
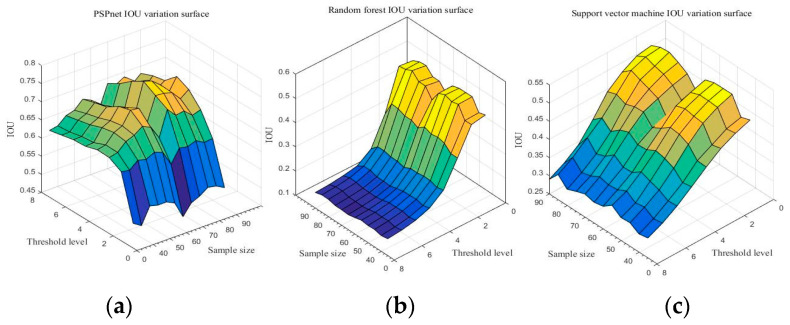
Accuracy of the three segmentation methods under the different sampling scales and threshold levels: (**a**) PSPnet, (**b**) RF, and (**c**) SVM.

Due to the comparison of the first sections, the PSPnet deep semantic segmentation network has obvious advantages over traditional machine learning in image quality and the IoU results of resampling. With sampling size *S* and threshold level T as independent variables, and IoU as the dependent variable, the IoU change surfaces of three segmentation methods are shown in Figure 10. (Detailed data are shown in Table A13, Table A14 and Table A15 of Appendix A).

**Figure 11:**
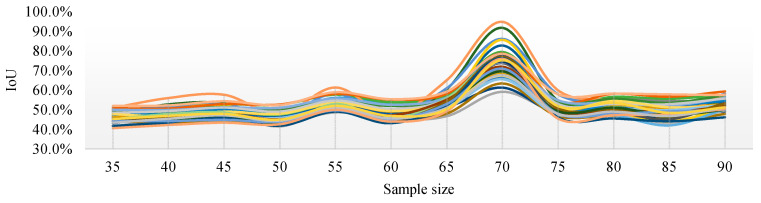
IoU curves of 40 test images with different sampling sizes when the threshold level T = 5.

Figure 10 shows the IoU changes of the three segmentation methods under different parameters. From Figure 10a, regardless of the sampling size *S*, the threshold level T = 5 can achieve better IoU. By selecting the threshold level T = 5, the performance of the IoU of PSPnet on 40 test sets after processing with different sampling sizes is shown in Figure 11 (each color curve represents a different test chart).

**Figure 12:**
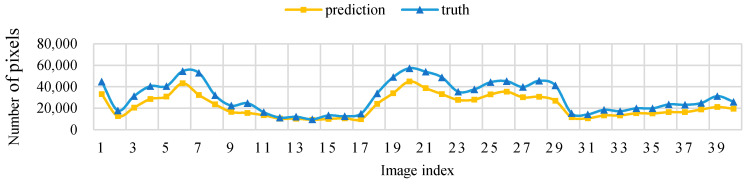
Threshold level T = 5, sampling size *S* = 70 pixels, test set image true value pixel, and the predicted pixel number change curve.

Figure 11 shows that, when the threshold level T = 5, the sampling parameters used to obtain a high IoU are mainly concentrated around the sampling size *S* = 70. Figure 12 shows the change curve of the true value and the predicted value of the 40 pictures in the test set when the sampling size is *S* = 70 and T = 5.

## 4. Discussion

### 4.1. Advantages and Limitations of the Model

In this study, an effective model (PLD-M) for kiwifruit orchard data acquisition from low-altitude UAV remote sensing images is proposed. This includes the detection and segmentation of kiwifruit vines, and the prediction of the possible canopy distribution. The use of a deep learning method increased the quality of the segmentation image, and intersection and union ratio, compared to those of traditional machine learning. The existing methods of dense tree canopy recognition and extraction can only obtain the number of fruit trees, canopy parameters, and other information. In contrast, the PLD-M model can not only count and locate kiwifruit plants, but also extract the vine distribution and possible canopy distribution of each plant. Compared with the apple branch extraction model proposed by J Wu et al. (2020), PLD-M can not only obtain the specific distribution of vines and the position of plants, but also extract the distribution density gradient of liana plants. The distribution range of the canopy is more refined, and the hollow area of the canopy caused by the external polygon of branches is avoided.

Due to the small size of the ground stump, camera lens distortion, and pan tilt stability, small distortions appear in the stitching process, which may cause tiny inconsistencies in the texture of different regions. In subsequent research, these problems could be addressed by improving the resolution of the remote sensing camera, using a lens with less distortion, and enhancing the pan tilt stability.

In this paper, we do not discuss the gap between the proposed PSPnet and other deep learning networks, such as that of Lucas Prado Osco et al. [28]. This is because, compared with the recognition of large areas, the mAP of deep learning network is not superior to traditional machine learning in the classification of kiwifruit vines. However, this study can optimize the data set and network input through feature fusion [29] and image fusion [30], and thus allow comparison of the differences in different neural networks in multi-feature and multi-channel images.

### 4.2. Research Significance and Prospect

After artificial pruning of kiwifruit plants, the originally dense vines show an uneven and sparse distribution. Although the sparse degree of vine distribution of a single plant is within the reasonable range of artificial control, the vine distribution among kiwifruit plants is uneven. This may lead to the overlap of the canopy between kiwifruit plants, thus affecting plant photosynthesis and, ultimately, yield. The orchard information is collected using a low-altitude UAV and input into PLD-M to obtain the distribution of vines of each plant and the distribution of vines among plants, thus allowing evaluation of the rationality of winter pruning and assisting in decision making. This can not only be used for the evaluation of pruning, but also for variable rate spraying and variable rate fertilization.

Possible future directions exist for this research. First, the model could be applied to the embedded system of UAVs for real-time orchard condition analysis, and thus to precision agriculture. Second, because the training of deep learning networks depends on Big Data, datasets should be further expanded and enriched in further study of the model. Third, for variable applications, models should be designed that can provide a greater amount of information, such as the elevation and overlap. Fourth, at present, the model is only suitable for kiwifruit plants, and future research can extend the model to other plants. Finally, the image information that is obtained by a visible light sensor is limited. To expand the amount of information that is collected, multispectral data will be introduced in future investigations.

## 5. Conclusions

In this study, an effective model (PLD-M) for kiwifruit orchard data acquisition from low-altitude UAV remote sensing images is proposed. This includes the detection and segmentation of kiwifruit vines, and prediction of the possible canopy distribution. The model is based on the YOLOv3 target recognition network and the PSPnet deep semantic segmentation network. The former extracts the trunk position of the kiwifruit plants and provides the location information of each plant, whereas the latter extracts the kiwifruit vines from the background. Then, the segmented image is input into a model to predict the canopy range using a resampling operation. Finally, the kiwifruit plant information, such as the vine position and canopy distribution, can be obtained from the low-altitude UAV images. To train and optimize the proposed model, this study constructed a training set that contained 300 labeled images to optimize and train the YOLOv3 network. We also constructed a training set that contained 300 labeled images to train and optimize the PSPnet deep semantic segmentation network that was used to extract the kiwifruit vines. To verify and optimize the accuracy of the canopy prediction that was obtained by PSPnet and resampling, another 40 high-definition images were selected from the UAV remote sensing images for manual truth labeling. The results showed that the canopy data that were predicted by the model were close to the artificial data (the IoU of the canopy distribution was between 70% and 95%). Therefore, the proposed model can accurately extract the vine distribution and plant position of each plant in a kiwifruit orchard, based on UAV data, allowing rapid, non-destructive, and stable monitoring of kiwifruit plants.

## Figures and Tables

**Table 1:** Aerial photography and image parameter.

Route Parameters	Detailed Data
flight altitude	20 m
Heading/sideward overlap	80%
Image size	5472 × 3648
Image channel	RGB
Imaging band	R (700 mm) G (546.1 mm) B (435.8 mm)

**Table 2:** Training environment and equipment.

Device	Version
Programming framework	pytorch1.7.1
Programing language	Python3.6.10
GPU	NVIDIA-GeForce RTX3080s-OC-10GB
CPU	Inter-Xeon E5-2600-8-core processor

**Table 3:** PSPnet training hyper parameters and the formula.

Hyper Parameter	Value
Epoch	100
Batch_size	8, 4
Learning_rate	$10^{-4}$, $10^{-5}$
Loss_function	cross entropy loss

**Table 4:** Block color and category correspondence.

Resample Area Color	Classes
Dark green	Core area
Green	Coverage area
White	Transition area
Brown	Naked land

**Table 5:** Segmented image evaluation.

Method	mAP	SNR
RF	75.26%	7.2498
SVM	85.8%	10.5154
PSPnet	71.2%	12.9523

**Table 6:** Threshold level and the threshold correspondence.

Threshold Level: T	1	2	3	4	5	6	7	8
Threshold	0.88	0.77	0.66	0.55	0.44	0.33	0.22	0.11

## Data Availability

Publicly available datasets were analyzed in this study. This data can be found here: [https://github.com/eletricsheep/PLD-M/tree/main].

## Appendix A

**Table A1:** The IoU of sample size *S* = 35 varies with the threshold level T.

Methods	T = 1	T = 2	T = 3	T = 4	T = 5	T = 6	T = 7	T = 8
PSPnet	0.4969	0.6192	0.6380	0.6394	0.6292	0.6271	0.6197	0.6126
RF	0.4742	0.4642	0.3559	0.2531	0.2084	0.1877	0.1764	0.1704
SVM	0.4743	0.4604	0.4408	0.4119	0.3800	0.3494	0.3243	0.3056

**Table A2:** The IoU of sample size *S* = 40 varies with the threshold level T.

Methods	T = 1	T = 2	T = 3	T = 4	T = 5	T = 6	T = 7	T = 8
PSPnet	0.4824	0.6152	0.6440	0.6447	0.6311	0.6279	0.6200	0.6172
RF	0.4658	0.4630	0.3560	0.2463	0.2011	0.1828	0.1720	0.1660
SVM	0.4658	0.4561	0.4344	0.4132	0.3752	0.3471	0.3203	0.3006

**Table A3:** The IoU of sample size *S* = 45 varies with the threshold level T.

Methods	T = 1	T = 2	T = 3	T = 4	T = 5	T = 6	T = 7	T = 8
PSPnet	0.5283	0.6341	0.6657	0.6705	0.6640	0.6517	0.6443	0.6370
RF	0.5136	0.5121	0.3809	0.2562	0.2093	0.1896	0.1792	0.1732
SVM	0.5132	0.4992	0.4780	0.4421	0.3988	0.3611	0.3362	0.3155

**Table A4:** The IoU of sample size *S* = 50 varies with the threshold level T.

Methods	T = 1	T = 2	T = 3	T = 4	T = 5	T = 6	T = 7	T = 8
PSPnet	0.5225	0.6287	0.6750	0.6808	0.6751	0.6629	0.6545	0.6435
RF	0.5135	0.5052	0.3882	0.2570	0.2069	0.1883	0.1775	0.1717
SVM	0.5129	0.5011	0.4810	0.4537	0.4096	0.3692	0.3317	0.3135

**Table A5:** The IoU of sample size *S* = 55 varies with the threshold level T.

Methods	T = 1	T = 2	T = 3	T = 4	T = 5	T = 6	T = 7	T = 8
PSPnet	0.5230	0.6402	0.6914	0.7158	0.7061	0.6846	0.6761	0.6658
RF	0.5130	0.5150	0.3831	0.2528	0.2064	0.1882	0.1788	0.1755
SVM	0.5125	0.5014	0.4856	0.4565	0.4150	0.3789	0.3483	0.3310

**Table A6:** The IoU of sample size *S* = 60 varies with the threshold level T.

Methods	T = 1	T = 2	T = 3	T = 4	T = 5	T = 6	T = 7	T = 8
PSPnet	0.5119	0.6318	0.6801	0.7128	0.6946	0.6756	0.6616	0.6550
RF	0.5051	0.5040	0.3944	0.2530	0.2000	0.1814	0.1722	0.1685
SVM	0.5044	0.4920	0.4773	0.4382	0.4012	0.3612	0.3356	0.3123

**Table A7:** The IoU of sample size *S* = 65 varies with the threshold level T.

Methods	T = 1	T = 2	T = 3	T = 4	T = 5	T = 6	T = 7	T = 8
PSPnet	0.4660	0.5905	0.6434	0.6655	0.6713	0.6492	0.6306	0.6229
RF	0.4581	0.4595	0.3728	0.2396	0.1923	0.1760	0.1681	0.1630
SVM	0.4581	0.4541	0.4268	0.4237	0.3992	0.3597	0.3331	0.3074

**Table A8:** The IoU of sample size *S* = 70 varies with the threshold level T.

Methods	T = 1	T = 2	T = 3	T = 4	T = 5	T = 6	T = 7	T = 8
PSPnet	0.4959	0.6269	0.7134	0.7313	0.7506	0.7305	0.7039	0.6844
RF	0.4891	0.4986	0.3868	0.2474	0.1943	0.1772	0.1697	0.1651
SVM	0.4891	0.4795	0.4656	0.4507	0.4171	0.3773	0.3389	0.3139

**Table A9:** The IoU of sample size *S* = 75 varies with the threshold level T.

Methods	T = 1	T = 2	T = 3	T = 4	T = 5	T = 6	T = 7	T = 8
PSPnet	0.5063	0.6228	0.6888	0.7293	0.7432	0.7221	0.6961	0.6732
RF	0.5042	0.5148	0.3968	0.2512	0.1954	0.1799	0.1719	0.1679
SVM	0.5042	0.4941	0.4756	0.4491	0.4073	0.3575	0.3256	0.3050

**Table A10:** The IoU of sample size *S* = 80 varies with the threshold level T.

Methods	T = 1	T = 2	T = 3	T = 4	T = 5	T = 6	T = 7	T = 8
PSPnet	0.5221	0.6311	0.6926	0.7230	0.7248	0.7167	0.6931	0.6863
RF	0.5173	0.5259	0.3982	0.2539	0.2032	0.1833	0.1774	0.1724
SVM	0.5162	0.5000	0.4844	0.4401	0.3870	0.3482	0.3187	0.2949

**Table A11:** The IoU of sample size *S* = 85 varies with the threshold level T.

Methods	T = 1	T = 2	T = 3	T = 4	T = 5	T = 6	T = 7	T = 8
PSPnet	0.5177	0.6180	0.6722	0.6981	0.6938	0.6846	0.6674	0.6590
RF	0.5150	0.5134	0.4009	0.2536	0.2024	0.1843	0.1771	0.1730
SVM	0.5136	0.5093	0.4946	0.4511	0.4084	0.3764	0.3444	0.3192

**Table A12:** The IoU of sample size *S* = 90 varies with the threshold level T.

Methods	T = 1	T = 2	T = 3	T = 4	T = 5	T = 6	T = 7	T = 8
PSPnet	0.5052	0.6204	0.6798	0.7153	0.7309	0.6998	0.6929	0.6794
RF	0.5022	0.5091	0.3409	0.2266	0.1857	0.1751	0.1681	0.1650
SVM	0.5022	0.4958	0.4735	0.4420	0.3875	0.3399	0.3112	0.2901

**Table A13:** The IoU of PSPnet varies with sampling size *S* and threshold level T.

		***S* = 35**	***S* = 40**	***S* = 45**	***S* = 50**	***S* = 55**	***S* = 60**
	
T = 1		0.4969	0.4824	0.5283	0.5225	0.5230	0.5119
T = 2		0.6192	0.6152	0.6341	0.6287	0.6402	0.6318
T = 3		0.6380	0.6440	0.6657	0.6750	0.6914	0.6801
T = 4		0.6394	0.6447	0.6705	0.6808	0.7158	0.7128
T = 5		0.6292	0.6311	0.6640	0.6751	0.7061	0.6946
T = 6		0.6271	0.6279	0.6517	0.6629	0.6846	0.6756
T = 7		0.6197	0.6200	0.6443	0.6545	0.6761	0.6616
T = 8		0.6126	0.6172	0.6370	0.6435	0.6658	0.6550
		***S* = 65**	***S* = 70**	***S* = 75**	***S* = 80**	***S* = 85**	***S* = 90**
	
T = 1		0.4660	0.4959	0.5063	0.5221	0.5177	0.5052
T = 2		0.5905	0.6269	0.6228	0.6311	0.6180	0.6204
T = 3		0.6434	0.7134	0.6888	0.6926	0.6722	0.6798
T = 4		0.6655	0.7313	0.7293	0.7230	0.6981	0.7153
T = 5		0.6713	0.7506	0.7432	0.7248	0.6938	0.7309
T = 6		0.6492	0.7305	0.7221	0.7167	0.6846	0.6998
T = 7		0.6306	0.7039	0.6961	0.6931	0.6674	0.6929
T = 8		0.6229	0.6844	0.6732	0.6863	0.6590	0.6794

**Table A14:** The IoU of RF varies with sampling size *S* and threshold level T.

		***S* = 35**	***S* = 40**	***S* = 45**	***S* = 50**	***S* = 55**	***S* = 60**
	
T = 1		0.4742	0.4658	0.5136	0.5135	0.5130	0.5051
T = 2		0.4642	0.4630	0.5121	0.5052	0.5150	0.5040
T = 3		0.3559	0.3560	0.3809	0.3882	0.3831	0.3944
T = 4		0.2531	0.2463	0.2562	0.2570	0.2528	0.2530
T = 5		0.2084	0.2011	0.2093	0.2069	0.2064	0.2000
T = 6		0.1877	0.1828	0.1896	0.1883	0.1882	0.1814
T = 7		0.1764	0.1720	0.1792	0.1775	0.1788	0.1722
T = 8		0.1704	0.1660	0.1732	0.1717	0.1755	0.1685
		***S* = 65**	***S* = 70**	***S* = 75**	***S* = 80**	***S* = 85**	***S* = 90**
	
T = 1		0.4581	0.4891	0.4891	0.5173	0.5150	0.5022
T = 2		0.4595	0.4986	0.4986	0.5259	0.5134	0.5091
T = 3		0.3728	0.3868	0.3868	0.3982	0.4009	0.3409
T = 4		0.2396	0.2474	0.2474	0.2539	0.2536	0.2266
T = 5		0.1923	0.1943	0.1943	0.2032	0.2024	0.1857
T = 6		0.1760	0.1772	0.1772	0.1833	0.1843	0.1751
T = 7		0.1681	0.1697	0.1697	0.1774	0.1771	0.1681
T = 8		0.1630	0.1651	0.1651	0.1724	0.1730	0.1650

**Table A15:** The IoU of SVM varies with sampling size *S* and threshold level T.

		***S* = 35**	***S* = 40**	***S* = 45**	***S* = 50**	***S* = 55**	***S* = 60**
	
T = 1		0.4743	0.4658	0.5132	0.5129	0.5125	0.5044
T = 2		0.4604	0.4561	0.4992	0.5011	0.5014	0.4920
T = 3		0.4408	0.4344	0.4780	0.4810	0.4856	0.4773
T = 4		0.4119	0.4132	0.4421	0.4537	0.4565	0.4382
T = 5		0.3800	0.3752	0.3988	0.4096	0.4150	0.4012
T = 6		0.3494	0.3471	0.3611	0.3692	0.3789	0.3612
T = 7		0.3243	0.3203	0.3362	0.3317	0.3483	0.3356
T = 8		0.3056	0.3006	0.3155	0.3135	0.3310	0.3123
		***S* = 65**	***S* = 70**	***S* = 75**	***S* = 80**	***S* = 85**	***S* = 90**
	
T = 1		0.4581	0.4891	0.5042	0.5162	0.5136	0.5022
T = 2		0.4541	0.4795	0.4941	0.5000	0.5093	0.4958
T = 3		0.4268	0.4656	0.4756	0.4844	0.4946	0.4735
T = 4		0.4237	0.4507	0.4491	0.4401	0.4511	0.4420
T = 5		0.3992	0.4171	0.4073	0.3870	0.4084	0.3875
T = 6		0.3597	0.3773	0.3575	0.3482	0.3764	0.3399
T = 7		0.3331	0.3389	0.3256	0.3187	0.3444	0.3112
T = 8		0.3074	0.3139	0.3050	0.2949	0.3192	0.2901
